# Self-introduction of a two-meter nylon string into the urinary bladder: A case report^☆^^☆^

**DOI:** 10.1016/j.radcr.2022.01.010

**Published:** 2022-01-18

**Authors:** Firmantya Hadi Pranata, Yudhistira Pradnyan Kloping, Doddy Moesbadianto Soebadi

**Affiliations:** a Department of Urology, Faculty of Medicine, Universitas Airlangga, Surabaya, East Java, Indonesia; b Dr. Soetomo General-Academic Hospital, Surabaya, East Java, Indonesia

**Keywords:** ER, Emergency Room, CT, Computed Tomography, Foreign body, Urinary bladder, Bladder foreign body, Autoeroticism

## Abstract

A foreign body of the urinary bladder can be caused by several factors. Several patients deliberately insert foreign bodies via the urethra due to psychiatric issues to achieve sexual pleasure. Self-inserted urinary bladder foreign bodies remain a significant challenge in the urology field regarding diagnosis and management as patients may be late in seeking medical assistance due to guilt and embarrassment. We aimed to report a 37-year-old man who inserted a two-meter nylon string into his urethra for sexual gratification.

## Introduction

A foreign body of the urinary bladder is a relatively rare case, usually caused by self-insertion. However, the number of these cases has raised in the last few decades [1]. Items are inserted into the bladder through self-insertion, iatrogenic process, migration from adjacent organs, or trauma penetrating the bladder [2]. People who insert foreign bodies for sexual gratification may avoid seeking medical help due to embarrassment and guilt. Thus, oftentimes the patients are late to be admitted, potentially leading to serious complications, including recurrent urinary tract infection, stone formation, and urosepsis [3,4]. Urinary bladder foreign bodies remain a significant challenge for urologists as dishonest patients may hinder proper diagnosis and management. Proper management requires an understanding of the clinical and imaging characteristics of patients with bladder foreign bodies. In Indonesia, these cases are rarely reported, thus, we report a 37-year-old male patient with a self-inserted nylon string in his bladder.

## Case presentation

A 37-year-old male was admitted to the emergency room (ER) of Dr. Soetomo General-Academic Hospital with a chief complaint of lower abdominal pain two hours before being admitted. The patient inserted a long nylon string, used for beads, into his urethra. The patient inserted the string while being fully erect and watching a pornographic video. He also complained of hematuria and voiding difficulties. Communicating with the patient was challenging due to embarrassment and a history of speech difficulties. The patient admitted that this was the first time that he had done this, although he masturbated and consumed pornographic content daily. The patient is not married with no history of sexual intercourse. He lived alone with his mother, who was suffering from a mental disorder. The general and hemodynamic condition of the patient was stable. Physical examination showed suprapubic pain and tenderness. Other urological signs were negative. The distal end of the string was not visible in the external urethral orifice. Digital rectal examination showed normal results. Laboratory results showed slight leukocytosis (14.250 × 10^9^/L). Other parameters were normal.

## Investigations/Imaging findings

**Fig. 1:**
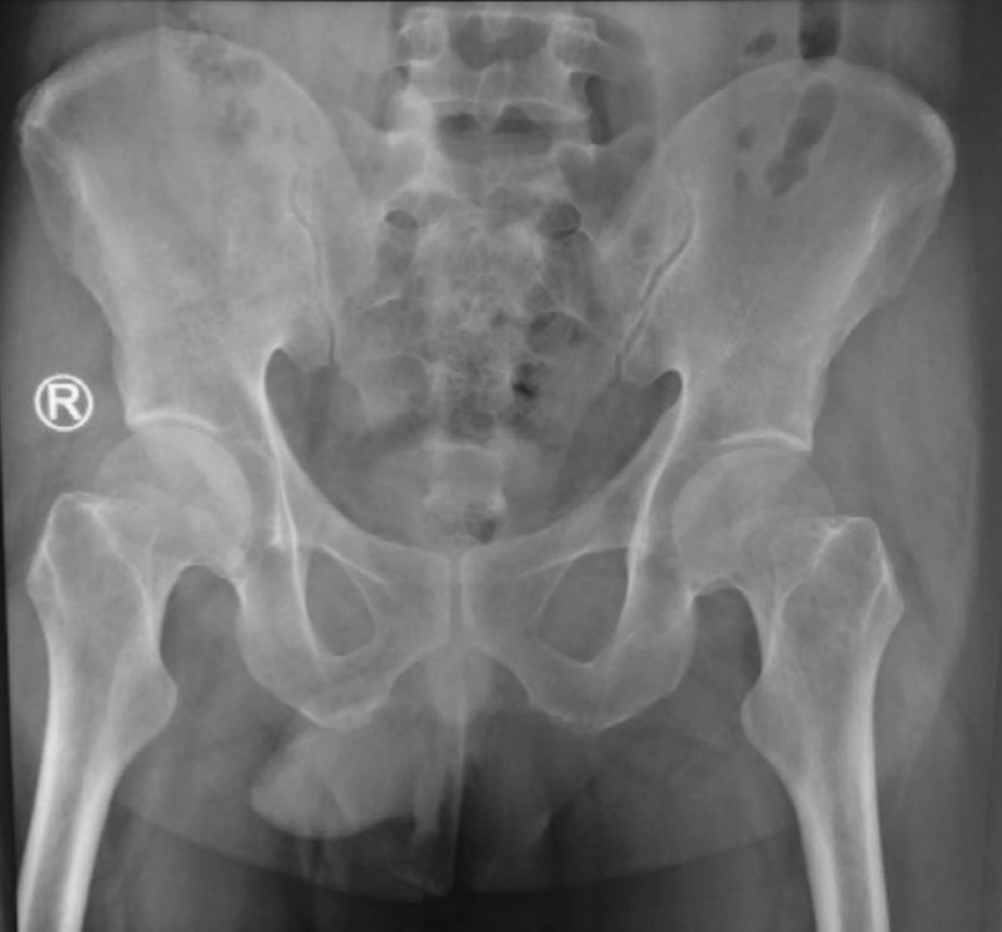
Plain abdominal X-Ray with a posteroanterior view and visible penis showed normal findings

**Fig. 2:**
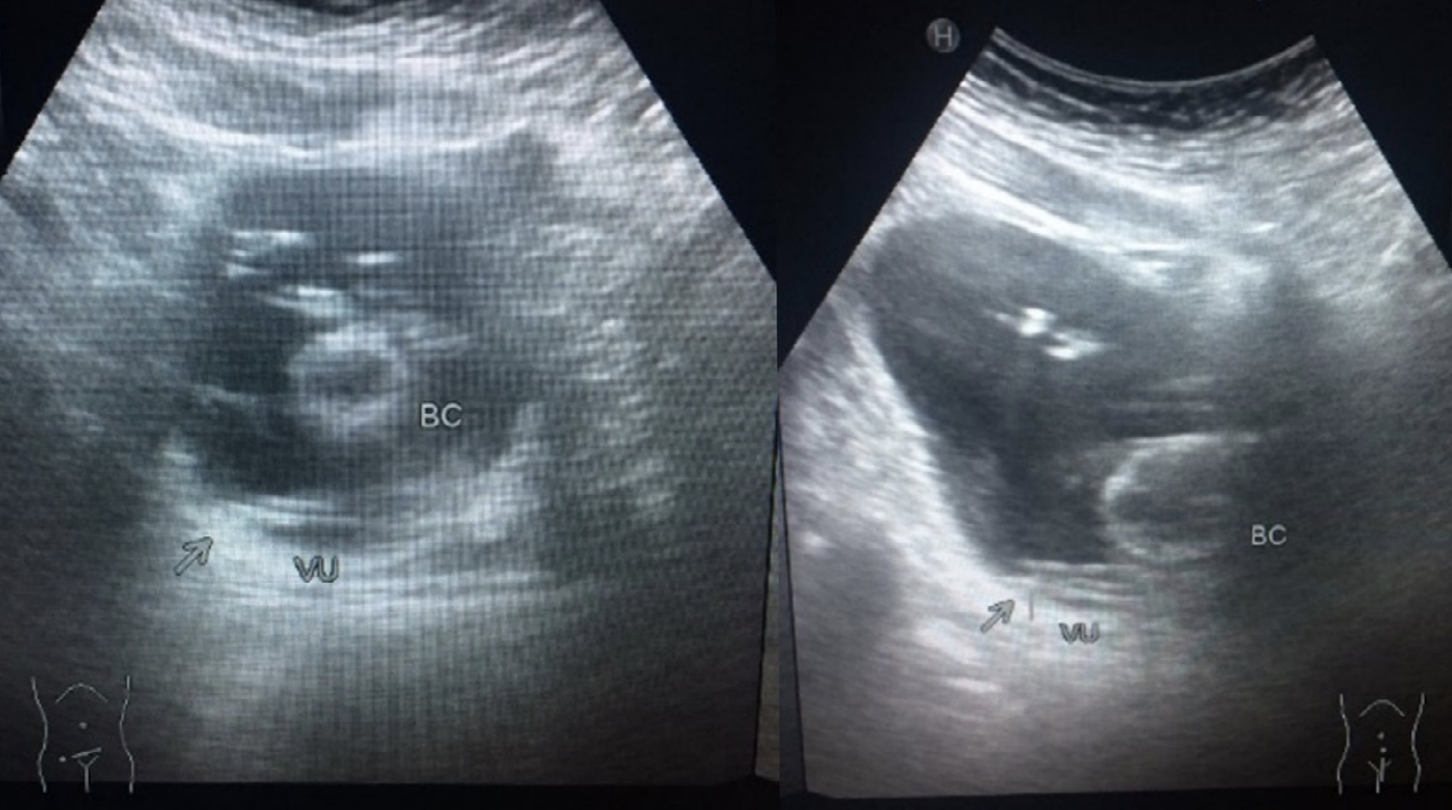
USG examination showed a hypoechoic appearance in the posterior area of the bladder

Plain abdominal X-Ray with a posteroanterior view and visible penis result was normal, as shown in Figure 1. A hypoechoic pattern was seen in a USG examination in Figure 2.

## Differential diagnosis

The patient was diagnosed with a urinary bladder foreign body based on history taking and imaging results.

## Treatment

**Fig. 3:**
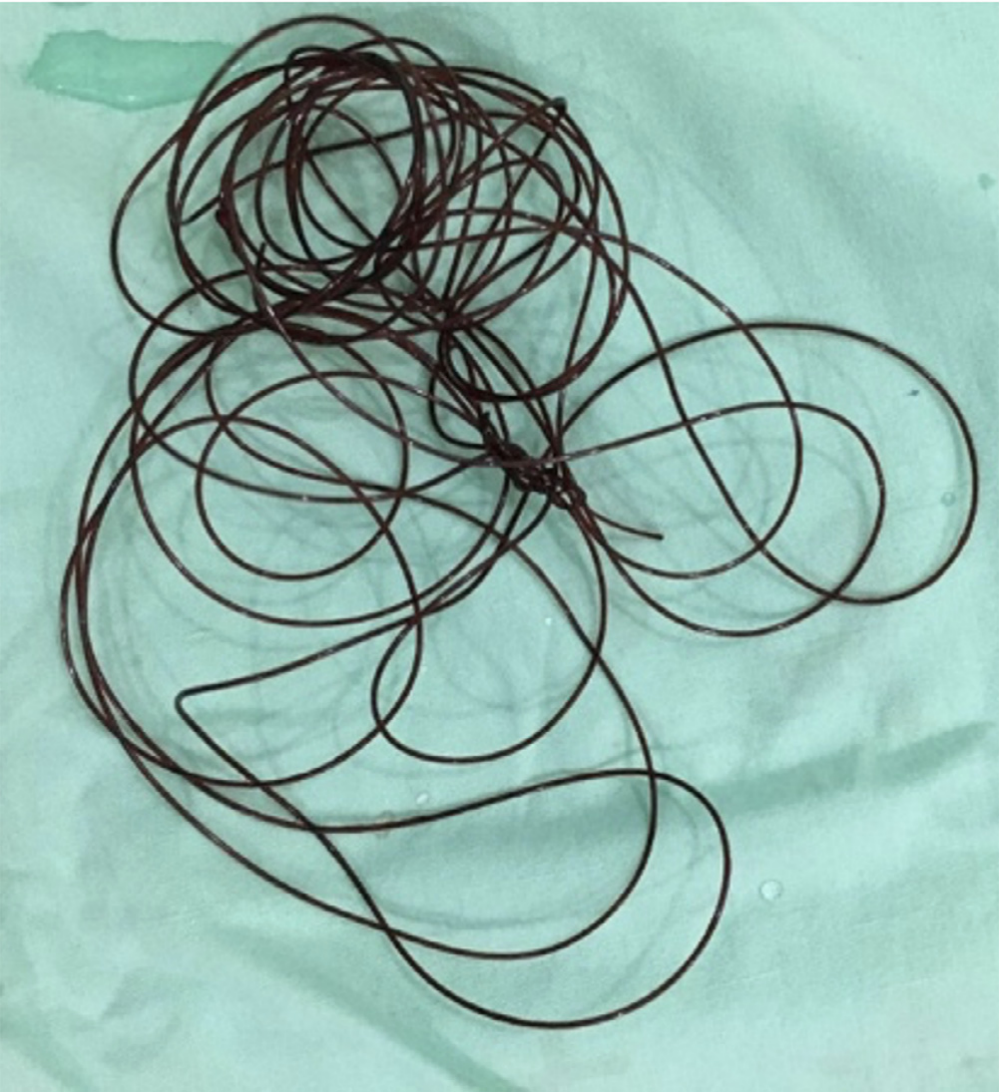
A two-meter nylon string was evacuated from the patient's bladder

A 20 Fr urinary catheter was inserted with hopes that active irrigation may help the passage of the string. A 0.9% Natrium Chloride installation was used for irrigation, resulting in blood clots, however, the string was unable to pass. Therefore, cystoscopy with local anesthesia was performed. A black bundle of nylon string was seen during the procedure. The evacuation was carried out using grasping forceps. A two-meter nylon string was extracted from the patient's bladder, as shown in Figure 3. The patient's postoperative complaints, vital signs, and urine production were within normal limits.

## Outcome and follow-up

The patient's postoperative complaints, vital signs, and urine production were within normal limits. The patient was then discharged two days after the operation and came to the outpatient with no further complaints. He was also referred to the psychiatric department's outpatient unit for further evaluation regarding his sexual behavior and was assessed with obsessive-compulsive disorder, with predominant obsessive tendencies and paraphilia.

## Discussion

Even though they are rare, the urinary bladder is the most common site of foreign bodies in the genitourinary tract. A variety of conceivable items have been inserted into the bladder, including hairpins, hair clips, straws, matchsticks, pencils, and even worms [5], [6], [7]. There are several reasons behind the insertion of this foreign body such as psychiatric problems, drug abuse, misconduct, and sexual satisfaction. Most of the time, the reason for foreign body insertion was due to autoerotic manipulation. A psychiatric disorder was also common in this patient as mostly the patient possesses schizoid personality, borderline personality, and sexual exotic impulse [8]. In several cases, patients may deny inserting objects and claimed that the items were accidentally inserted instead. This is due to the feeling of embarrassment of admitting his behavior to achieve sexual gratification, which often leads to difficulties in acquiring accurate information during history taking. Therefore, patients often came to the doctor with late and serious complications [9]. The patient in this report also hesitated to share his condition during history taking due to embarrassment. Aside from being embarrassed, he was also difficult to communicate with. A psychiatric evaluation showed that the patient was suffering from an obsessive-compulsive disorder, with predominant obsessive tendencies and paraphilia. Sexual gratification achieved by the self-insertion of objects into the urethra is classified as a paraphilia and a combination of sadomasochism and fetishistic behavior [10]. Aside from erotic stimulation, self-insertion can be done by psychotic patients, mentally retarded people, and children out of curiosity. Previous studies reported that urethral manipulation is a form of paraphilia and sadomasochistic with a fetishistic aspect. This activity is also suggested as a form of self-punishment and can increase the likelihood of suicide. Therefore, psychiatric consultation was recommended for these patients. The patient complained of lower abdominal pain, which is consistent with the reports of many previous studies. Short-term symptoms and signs of genitourinary tract foreign bodies include pain in the surrounding area of the item, dysuria, hematuria, weak stream, and urinary retention. The symptoms are due to the irritation of the object on the bladder's mucosa and the reduced capacity of the bladder. Trauma on the bladder wall causes hematuria [11], [12], [13]. If left untreated, serious complications such as stone formation, recurrent infection, and sepsis may occur [10]. In a suspicion toward the foreign body in the bladder, USG and plain abdominal X-Ray are the first imaging modalities that should be recommended. Plain radiography was used to assess the presence of radiopaque or radiolucent objects inside the bladder. Ultrasound can locate the object inside the bladder and may be beneficial in the visualization of radiolucent objects. In urethral or bladder rupture cases, urethrography or computed tomography (CT) cystography may be recommended [14]. Radiological examinations are performed to determine the size, position, and number of the items. Possible abnormalities of the bladder must also be considered before planning an intervention [2,15]. The main principle in the management of foreign body in the bladder was object evacuation without causing injury in the bladder and urethra and to overcome complications. The size and shape of the object are included in the consideration in the choice of therapy. The small-sized object can be extracted directly without prior fragmentation while the large-sized object needs to be fragmented first. If traumatic damage to the bladder was expected, an endoscopic approach is preferred [8]. Most items can be removed transurethrally using cystoscopy grasping forceps. Cystoscopy procedure works as both a diagnostic tool and means of treatment. During the extraction, urethral injuries must be avoided. Knotting is also a possible complication [16]. Treatment decision is based on the size and shape complexity of the foreign body to prevent iatrogenic injuries [17,18]. Cystoscopy is recommended for small objects, whereas large impacted objects may require open removal via suprapubic cystostomy [9]. In some cases, irrigation may also be attempted for a non-invasive approach [19]. In this case, however, irrigation was unsuccessful due to the complexity of a nylon string.

## Conclusion

A careful and proper evaluation of a patient's complete history combined with imaging modalities is necessary to assess a foreign body in the urinary tract. Total removal and complete clearance are the main principles in the management of this case to avoid the risk of further bladder injury. Cystoscopy is recommended for both diagnosing and extracting the foreign body. Further psychiatric evaluation is necessary to prevent reoccurrences.

## Author contributions

FHP, YPK, and DMS contributed equally to this article. All authors have read the manuscript and agreed to the contents.

## Patient consent and ethical approval

Informed consent for patient information to be published in this article was obtained. Appropriate informed consent was obtained for the publication of this case report and accompanying images. This report has been approved by the ethical committee of Dr. Soetomo General-Academic Hospital.
